# Viral etiology and seasonal trends of pediatric acute febrile illness in southern Puerto Rico; a seven-year review

**DOI:** 10.1371/journal.pone.0247481

**Published:** 2021-02-19

**Authors:** Liliana Sánchez-González, Talia M. Quandelacy, Michael Johansson, Brenda Torres-Velásquez, Olga Lorenzi, Mariana Tavarez, Sanet Torres, Luisa I. Alvarado, Gabriela Paz-Bailey

**Affiliations:** 1 Dengue Branch, Centers for Disease Control and Prevention, CDC, San Juan, Puerto Rico; 2 Saint Luke’s Episcopal Hospital Consortium, Ponce Health Sciences University, Ponce, Puerto Rico; Fundacao Oswaldo Cruz, BRAZIL

## Abstract

**Background:**

Acute febrile illness (AFI) is an important cause for seeking health care among children. Knowledge of the most common etiologic agents of AFI and its seasonality is limited in most tropical regions.

**Methodology/Principal findings:**

To describe the viral etiology of AFI in pediatric patients (≤18 years) recruited through a sentinel enhanced dengue surveillance system (SEDSS) in Southern Puerto Rico, we analyzed data for patients enrolled from 2012 to May 2018. To identify seasonal patterns, we applied time-series analyses to monthly arboviral and respiratory infection case data. We calculated coherence and phase differences for paired time-series to quantify the association between each time series.

A viral pathogen was found in 47% of the 14,738 patients. Influenza A virus was the most common pathogen detected (26%). The incidence of Zika and dengue virus etiologies increased with age. Arboviral infections peaked between June and September throughout the times-series. Respiratory infections have seasonal peaks occurring in the fall and winter months of each year, though patterns vary by individual respiratory pathogen.

**Conclusions/Significance:**

Distinct seasonal patterns and differences in relative frequency by age groups seen in this study can guide clinical and laboratory assessment in pediatric patients with AFI in Puerto Rico.

## Introduction

Globally, acute febrile illness (AFI) is a common reason for emergency department (ED) visits and an important cause of hospital admissions among children, with significant associated morbidity [1]. The etiology of pediatric AFI in Puerto Rico and the Caribbean includes diverse pathogens and conditions, including mosquito-borne and respiratory pathogens [2,3]. AFI can present non-specifically and varies greatly among different age groups, geographic regions, and seasons [4]. The diagnosis and treatment of AFI in children is challenging for health care providers, especially in places with limited diagnostic resources [5–7].

Viral infections are responsible for the majority of AFI in children within all age groups in different regions [4,8–10]. However, studies are not available for all ages or all geographical areas, and very few are conducted in South America and the Caribbean [5], where Puerto Rico, an unincorporated territory of the United States that has a tropical climate, is located [11,12]. A recent review on the epidemiology of AFI in Latin America identified only 17 studies in 8 countries, including patients of all ages, published in a 6-year period [12]. In this review, dengue virus (DENV) was the most common pathogen studied and reported, followed by chikungunya and Zika. Efforts to control DENV vectors, *Aedes* mosquitoes, have been unsuccessful; DENV and other arboviruses will likely continue to be important causes of AFI in the region [13].

Recent studies on fever etiology in tropical regions have limitations. For example, in most places, clinical samples are tested only for few specific pathogens, and some of the most common etiologies are not identified systematically [14,15]. For patients with influenza-like-illness (ILI), rhinovirus and influenza viruses are among the most common pathogens identified [16,17]. However, limited information is available on the contribution of various respiratory viruses to the AFI burden as few surveillance studies include a respiratory virus panel test [5,15,18].

Clinical management guidelines for pediatric AFI are usually not supported by information of the prevalent etiologic agents at a local level [19]. Early clinical suspicion based on knowledge of trends of common etiologies of AFI can improve the outcome of pediatric patients with an infection with potential life-threating complications like dengue, and better guide diagnostic approaches. Improved understanding influenza virus seasonality could help improve local vaccination strategies’ timing.

Previous reviews have found important gaps in the characterization of AFI etiology in children in tropical regions, including lack of testing for viral pathogens and short duration of studies to identify seasonality, and highlight the need for more comprehensive research [5,20].

We analyzed data from 2012–2018 from the Sentinel Enhanced Dengue Surveillance System (SEDSS), an expanded surveillance system for AFI in Ponce, Puerto Rico. We describe the viral etiology of AFI among pediatric patients, report differences in etiology patterns by age group, and identify and quantify seasonal trends of arboviruses and respiratory viruses’ epidemics.

## Materials and methods

SEDSS has been previously described [21,22]. Briefly, SEDSS is an ongoing AFI surveillance study in southern Puerto Rico. We used data from participants enrolled at Saint Luke’s Episcopal Hospital (SLEH) and the Outpatient Acute Care Clinic (CEMI for its initials in Spanish) in Ponce (SLEH from May 2012—December 2018 and CEMI from April 2016 –December 2018) and Guayama (SLEH Guayama- currently Menonite General Hospital, from February 2013—May 2015). SLEH Ponce is a tertiary level hospital that receives patients from Ponce and other municipalities in the southern Puerto Rico region.

Patients with fever (body temperature of ≥38.0°C (oral) or ≥38.5°C (axillary)) or reported history of fever during the previous 7 days, presenting to the ED or as a direct hospital admission, were eligible for enrollment. During June 2016 to June 2018, because of the Zika epidemic, patients with acute onset of generalized maculopapular rash, arthritis or arthralgia, or non-purulent conjunctivitis were eligible for participation, regardless of fever occurrence. Prior to enrollment, informed consent was obtained. The Centers for Disease Control and Prevention (CDC) and Ponce Medical School Foundation (PMSF) Institutional Review Boards approved the study protocol. Using a case investigation form (CIF), vital signs, signs and symptoms of current AFI, history of exposures and chronic disease, and clinical laboratory results were recorded at enrollment. All study participants had blood (5 mL in EDTA, 7 ml whole blood), urine (15 mL), and nasopharyngeal and oropharyngeal swabs collected at enrollment. Convalescent blood (5 mL in EDTA, 5 mL whole blood) and urine (10 mL) were collected at the follow-up visit or hospital discharge.

### Laboratory diagnostics

Molecular diagnostic testing was performed at CDC-Dengue Branch. Serum samples were tested using molecular and serological assays (RT-PCR and IgM Elisa) for dengue virus (DENV) 1–4 during the full study period, chikungunya virus (CHIKV) starting May 2014 and Zika virus (ZIKV) starting November 2015. Starting in 2012, nasopharyngeal and oropharyngeal swabs were tested by real time RT-PCR assay for influenza A and B viruses (IAV/IBV), and 12 other respiratory viruses (ORV) including adenovirus (AdV), human respiratory syncytial virus (HRSV), human metapneumovirus (HMPV), parainfluenza virus 1–4 (PIV 1–4), human rhinovirus HRV), and four human coronaviruses (HCoV 229E, OC43, NL63 and HKU1). We discontinued testing for HRV, PIV-2, PIV-4, and the four human coronaviruses after the first year as only 1 PIV-2, 41 HCoV infections (including 37 individual infections, and 4 co-infections), and no HRV or PIV-4 infections were identified.

### Definitions

A laboratory-positive DENV, CHIKV and ZIKV case had nucleic acid or IgM detected in a single specimen. A laboratory-positive IAV, IBV, HMPV, HRSV, AdV, PIV-1, PIV-2, PIV-3, PIV-4, HRV, and HCoV cases had the respective viral nucleic acid detected.

### Data analysis

We analyzed all pediatric (0-≤18 years) SEDSS data from May 7, 2012 to December 31, 2018 to describe demographic characteristics and to compare viral pathogens identification by sex and age group (0–23 months, 2–5 years, and 6–18 years). Binomial generalized linear models (GLM) with the log link were used to calculate relative risk (RR) with corresponding 95% confidence intervals (CI) to determine the association between age group and each virus detection. Statistical significance was defined as p < 0.05. Data were analyzed using R, version 3.1 (R Foundation for Statistical Computing) and R-Studio Integrated Development Environment for R (R-Studio, Inc).

To identify seasonal patterns, we applied time-series analyses to monthly arboviral and respiratory infection case data. We defined monthly total arboviral infections as the sum of confirmed dengue, chikungunya and Zika infections; and monthly combined respiratory infections as the sum of confirmed IAV, IBV, HMPV, HRSV, AdV, PIV-1, and PIV-3 occurring in each month. Total respiratory infections served as a proxy for ILI cases. We examined the seasonal trends of total arboviruses, as opposed to the individual arboviruses, because chikungunya and Zika case data were only available for at most two years and wavelet methods rely on longer time-series (i.e. >2 years) to identify seasonal trends. First, each case count time-series was log transformed (first adding 1 to all counts) and normalized (subtracting the mean of the time-series then dividing by the standard deviation). To extract the seasonal patterns of each time-series, normalized case count time-series were transformed into continuous wavelet transforms using Morlet wavelet decompositions (using the dplR R package) and filtered all components with a frequency in the range of 8 to 16 months, a common time-frame used to examine arboviral seasonality [23–26].

To quantify the association between each time series, we calculated coherence and phase differences for paired time-series [27]. Coherence is a metric of relatedness between two time-series at the same time-scale regardless of the temporal relationship between the two, where 1 indicates the series are identical in pattern and 0 indicates series have no similarity. We estimated the timing of the seasonal pattern for an individual pathogen using phase angles, and also estimated the lag (i.e. difference) in timing between two pathogens using the phase difference. The phase difference serves as a metric of similarity in timing of two time-series’ patterns relative to each other, i.e. if the patterns of two time-series are occurring at the same time or if one is occurring earlier or later in time (months) relative to the other. Average phase angle transforms for individual pathogens were estimated as the circular average of periodicity-specific phase angle (8–16 months) and was used to examine seasonal cycle in radians for each individual and total infections. Phase differences were derived as the pairwise difference in phase angles between two pathogens, as previously described [27–29]. Phase differences were calculated to assess the lag (in months) between time series. A phase difference of 0 indicated the two seasonal disease time-series are occurring at the same time (i.e. in phase), which may or may not have high coherence.

We compared total arboviral infections to total respiratory infections in coherence and phase analyses to quantify differences in seasonal patterns and timing of these two groups, using arboviruses as the reference. Individual respiratory infections were compared to total respiratory infections (reference group) to show how individual infection patterns compared to a proxy reflecting the ILI pattern. For easier reporting of findings, we use the Northern hemisphere definition for seasons (Winter: December, January and February; Spring: March, April and May; Summer: June, July and August and Fall: September, October and November).

## Results

Of 24,265 participants of all ages recruited during the study period, 14,751 patients were aged ≤18 years old. Thirteen participants had both insufficient blood and respiratory samples for testing and were excluded. Participants with insufficient blood samples but available respiratory samples results are included in the analysis for a total of 14,738 participants. Of participants 54% were male, median age was 4 years (IQR = 1–9), about two thirds (66%) were from Ponce, Guayama, and Juana Diaz municipalities, and median days post-illness onset (DPO) was 1.7 days (IQR = 1.3–2.8). Of participant children, 17% were hospitalized and 22% reported a chronic disease or condition with asthma being the most commonly reported (Table 1).

**Table 1:** Characteristics of pediatric patients, Sentinel Enhanced Dengue Surveillance System (SEDSS), Southern Puerto Rico, 2012–2018.

Demographics Characteristics and Clinical Presentation	Total n = 14,738	
**Age group**	**n**	**%**
0–23 months	4202	28.5
2–5 years	4505	30.6
6–18 years	6031	40.9
**Sex**	**n**	**%**
Male	7906	53.6
**Municipality of Residence**	**n**	**%**
Ponce	7423	50.4
Juana Diaz	687	4.7
Guayama	1547	10.5
Other	5081	34.5
**Days post-illness onset**	**n**	**%**
<3 days	11349	77.1
3–5 days	2946	20.0
6–8 days	430	2.9
**Disposition at enrollment**	**n**	**%**
Admitted	2568	17.4
Sent Home	12065	81.9
Transferred	86	0.6
Other	19	0.1
**Chronic disease or condition reported**	**n**	**%**
Asthma	2703	18.3
Diabetes mellitus	146	1.0
Immunodeficiency	33	0.2
Cancer	31	0.2
Sickle cell anemia	41	0.3
Other	240	1.6
None	11,544	78,4

**Fig 1:**
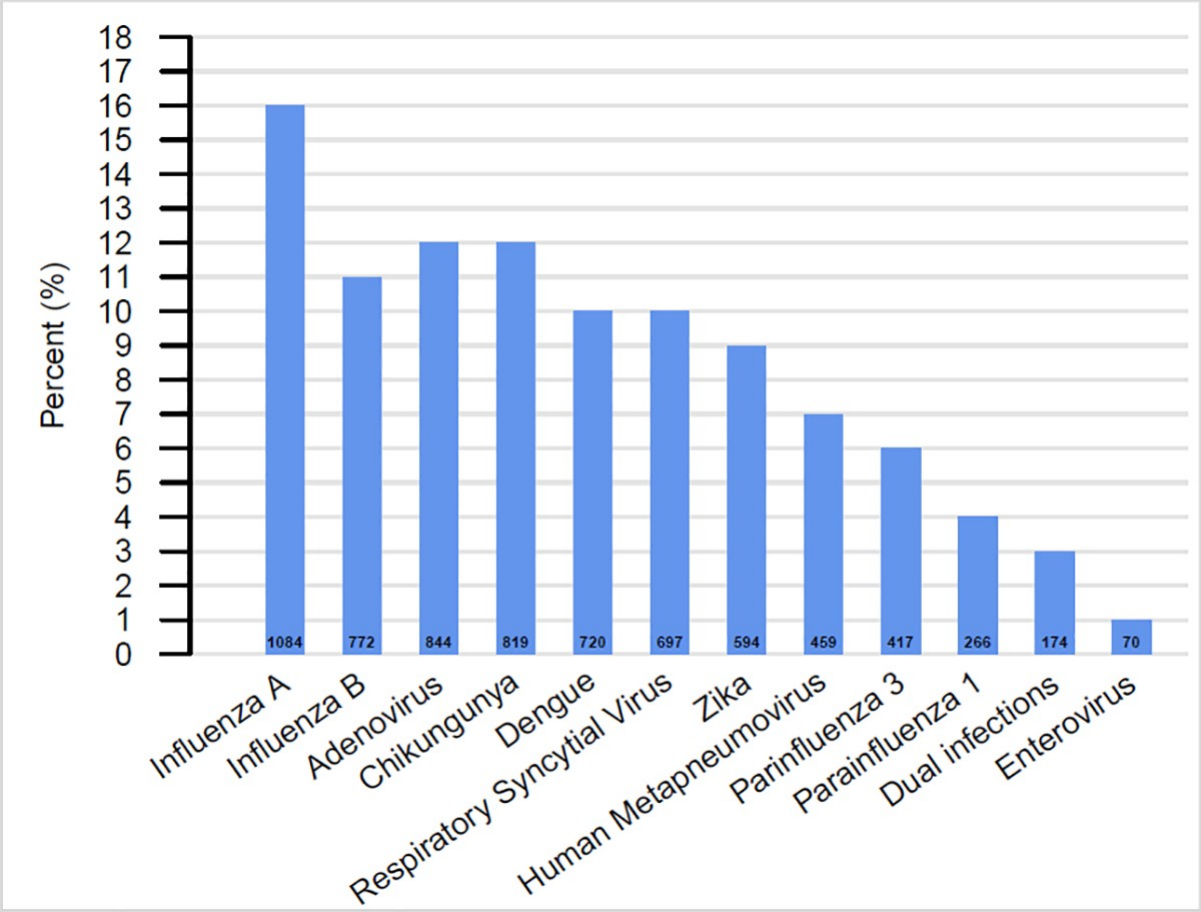
Overall distribution of viral etiology identified in pediatric patients, Sentinel Enhanced Dengue Surveillance System (SEDSS), Southern Puerto Rico, 2012–2018 (n = 6,916).

A viral pathogen was found in 47% (n = 6,916) of participants. Influenza was the most common pathogen identified (27%) with IAV identified in 16% (n = 1,084) of participants and IBV identified in 11% of participants (n = 772). The most common arbovirus identified was CHIKV in 12% of participants (n = 819), followed by DENV in 10% (n = 720) and ZIKV in 9% (n = 594) of participants. Overall, 174 (3%) participants had more than one pathogen identified (Fig 1). An etiologic pathogen was not identified in 52% of participants.

The study found differences in the frequency of respiratory pathogens by age. While IAV and IBV were more common in the older age groups, PIV 3 and HRSV were more frequent among youngest patients. The percentage of children diagnosed with AdV, PIV1, and HMPV was higher among children ≤5 years (Table 2). Positivity to arboviruses increased with age and all arboviruses were more likely to be diagnosed among older children (>5 years) (Table 2). DENV was identified in 1% of the children in the 0–23 months age group, and 2% (p = 0.123) of the 2–5 years age group. Among children >5 years old, DENV was identified in 10% of them (p <0.05, when compared to the 0–23 months age group). The difference in CHIKV identification was not statistically significant among the two youngest age groups, 5% and 6% among 0–23 months and 2–5 years, respectively (p = 0.284) but was significantly higher, 10%, among children >5 years old when compared to the 0–23 months age group (p<0.05). Following a similar trend, ZIKV was identified in 3%, 4%, and 13% of children in each group (p<0.005), respectively (Table 2).

**Table 2:** Etiologic diagnosis by age group, Sentinel Enhanced Dengue Surveillance System (SEDSS), Southern Puerto Rico, 2012–2018.

Pathogen	Age group												
	< 2 years			2 to 5 years			6–18 years^a^			Relative risk (RR) (2 to 5 compared to <2 as reference)	p value	Relative risk (RR) (6 to 18 compared to <2 as reference)	p value
	n	N	%	n	N	%	n	N	%				
Influenza A	182	4025	4.5	360	4280	8.4	542	5721	9.5	1.86 [1.57–2.22]	<0.001	2.1 [1.78–2.47]	<0.001
Influenza B	73	4025	1.8	173	4280	4.0	526	5721	9.2	2.23 [1.71–2.94]	<0.001	5.07 [4.01–6.51]	<0.001
Adenovirus	238	4025	5.9	388	4280	9.1	218	5721	3.8	1.53 [1.31–1.79]	<0.001	0.64 [0.54–0.77]	<0.001
PIV 1	86	4025	2.1	136	4280	3.2	44	5721	0.8	1.49 [1.14–1.95]	0.004	0.36 [0.25–0.51]	<0.001
PIV 3	239	4025	5.9	135	4280	3.2	43	5721	0.8	0.53 [0.43–0.65]	<0.001	0.13 [0.09–0.17]	<0.001
HRSV	343	4025	8.5	275	4280	6.4	79	5721	1.4	0.75 [0.65–0.88]	<0.001	0.16 [0.13–0.21]	<0.001
HMPV	157	4025	3.9	215	4280	5.0	87	5721	1.5	1.29 [1.05–1.58]	0.015	0.39 [0.3–0.5]	<0.001
Dengue	51	4132	1.2	74	4471	1.7	595	6019	9.9	1.34 [0.94–1.92]	0.123	8.01 [6.1–10.76]	<0.001
Chikungunya	169	3213	5.3	204	3462	5.9	446	4319	10.3	1.12 [0.92–1.37]	0.284	1.96 [1.66–2.34]	<0.001
Zika	61	2345	2.6	109	2576	4.2	424	3207	13.2	1.63 [1.2–2.23]	0.002	5.09 [3.94–6.68]	<0.001

Viral coinfections were identified in 174 patients. Most coinfections were of two respiratory viruses (n = 104, 60%): AdV and HRSV (n = 19), AdV and HMPV (n = 10), and AdV and IAV (n = 10). DENV infection and a respiratory virus infection was the second most common coinfection (n = 27, 16%).

### Evaluation of seasonal patterns

**Fig 2:**
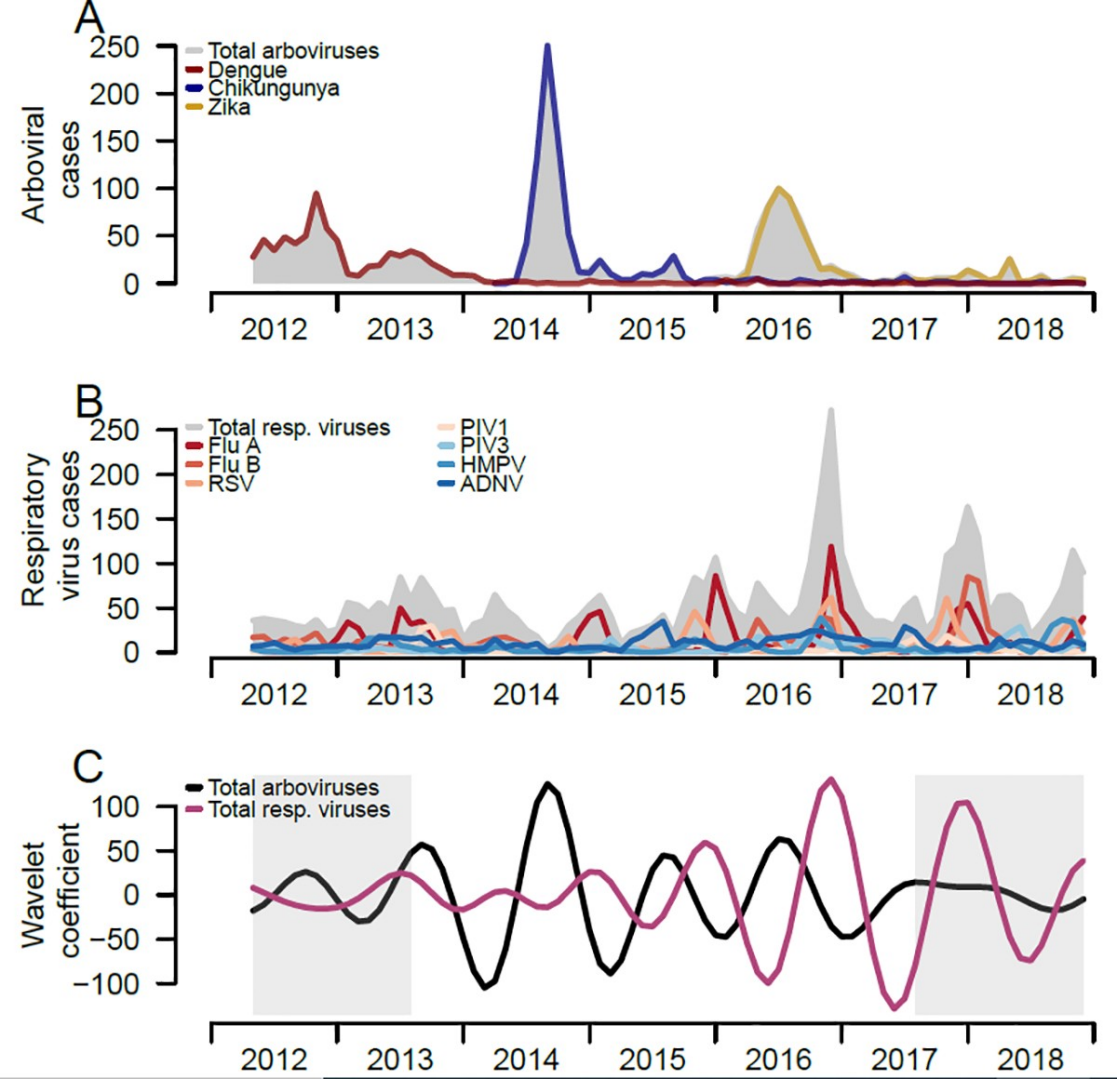
Trends of respiratory viruses and arboviruses among pediatric patients and comparison of both epidemic patterns, Sentinel Enhanced Dengue Surveillance System (SEDSS), Ponce, Puerto Rico, 2012–2018. A) Number of monthly RT-PCR confirmed respiratory viral infections overall and individually B) Number of monthly total and individual respiratory viral infections confirmed by RT-PCR. C) Reconstructed seasonal patterns of monthly total respiratory viral infections and arboviral infections using Morlet wavelet decomposition. Seasonal period was defined 8 to 16 months. Grey shading reflects the cone of influence, where the initial and last months of the seasonal time-series have more uncertainty (i.e. edge effects).

Overall, arboviral infections peaked between June and September over time, with major peaks corresponding to outbreaks of the individual arboviruses (Fig 2A). Respiratory infections have seasonal peaks occurring in the fall and winter months of each year, though patterns vary by individual respiratory pathogen (Fig 2B). The seasonal patterns of the two infection groups extracted from the wavelet decomposition also shows differing seasonality (Fig 2C), with seasonal peaks of respiratory infections occurring in the winter months and seasonal peaks of arboviral infections occurring in the late summer months.

**Fig 3:**
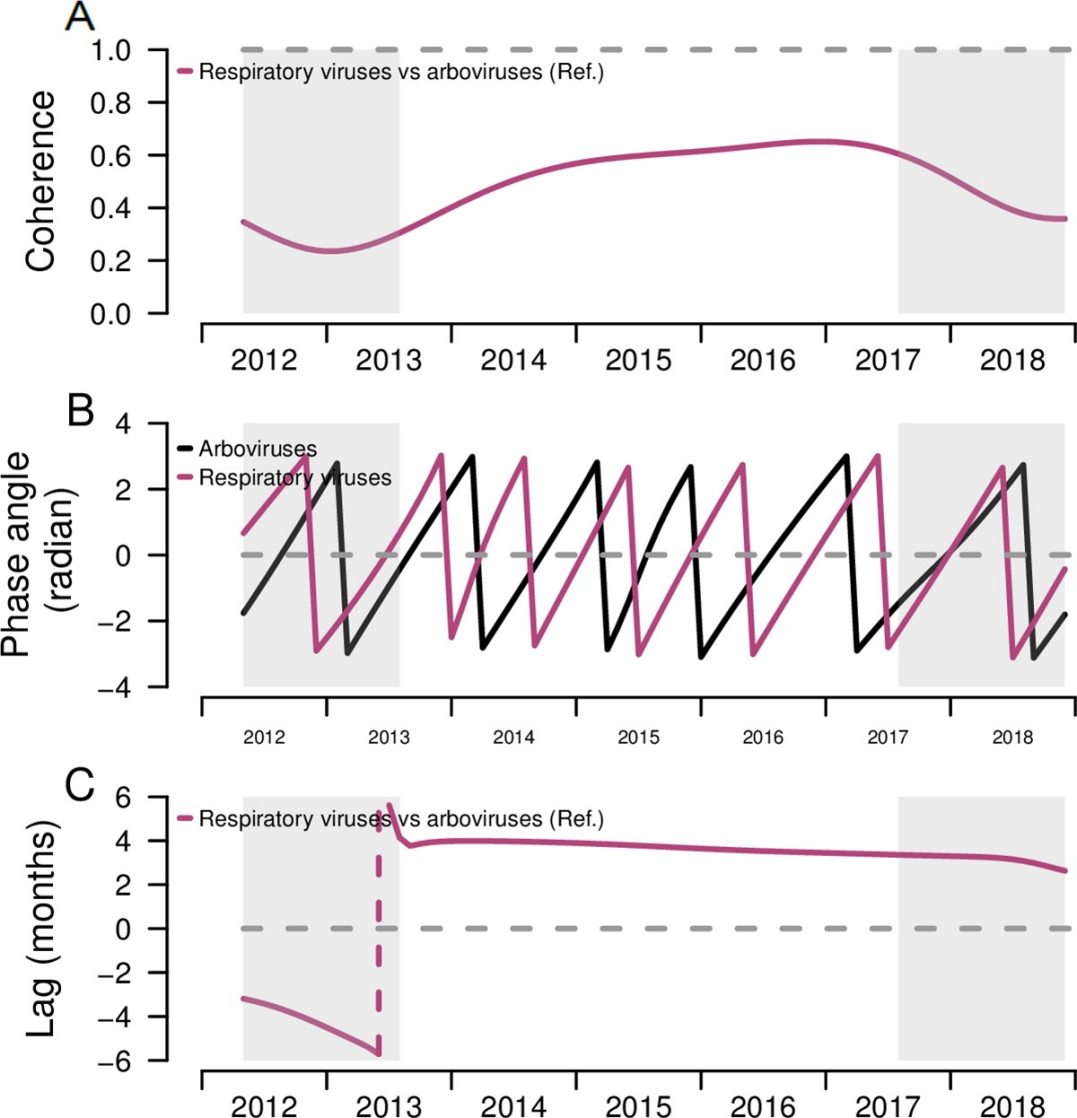
Coherence, phase angles, and lag of respiratory viruses and arboviruses among pediatric patients and comparison of both epidemic patterns, Sentinel Enhanced Dengue Surveillance System (SEDSS), Southern Puerto Rico, 2012–2018. A) Coherence comparing the similarity between the overall respiratory infection seasonal patterns to the arboviral infection patterns. Dashed line at 1 represents identical seasonal patterns and coherence at 0 refers to no association of two seasonal time-series. B) Phase angles of seasonal cycles of total respiratory infections and arboviral infections. C) Phase differences comparing the timing of respiratory infections seasonal cycles to arboviral infection epidemics. Dashed line at 0 refers to patterns of individual and overall respiratory infections occurring at the time (i.e. no difference in phase). Grey shading reflects the cone of influence, where edge-effects of the wavelet analysis influence the extraction and comparison of the season components. Negative values indicate the seasonal pattern of respiratory infections is behind arboviral infections, and positive values indicate the seasonal pattern of respiratory infections is ahead of arboviral infections.

To quantify differences in arboviruses and respiratory viruses’ seasonality, we estimated the pattern similarity and differences in timing between the seasonal time-series of total arboviruses and total respiratory viruses using coherence and phase analyses. The distinct seasonal patterns of the two infection groups observed in Fig 2A and 2B were confirmed through moderate estimates from the coherence analysis. The mean seasonal coherence between arboviruses and respiratory viruses was 0.48 (Table 3), reflecting similar, but not identical, seasonal patterns, in both pathogen types. Respiratory viruses occurred earlier in time compared to arboviruses, and the phase difference analysis found a 3.8 months difference in time between seasonality of arboviruses and respiratory viruses, further supporting differences in their seasonal timing (Fig 3B and 3C).

**Table 3:** Estimated overall mean seasonal coherence and phase difference (in months) of individual respiratory infections and arboviruses compared to all respiratory infections, Sentinel Enhanced Dengue Surveillance System (SEDSS), Southern Puerto Rico, 2012–2018.

Infection	Estimated coherence	Estimated lag
Influenza A	0.80	0.1 months
Influenza B	0.50	-1.0 months
Respiratory Syncytial Virus	0.59	1.0 months
Parainfluenza virus 1	0.40	2.4 months
Parainfluenza virus 3	0.51	-3.2 months
Human metapneumovirus	0.62	0.2 months
Adenovirus	0.32	-4.8 months
Arboviruses	0.48	3.8

**Fig 4:**
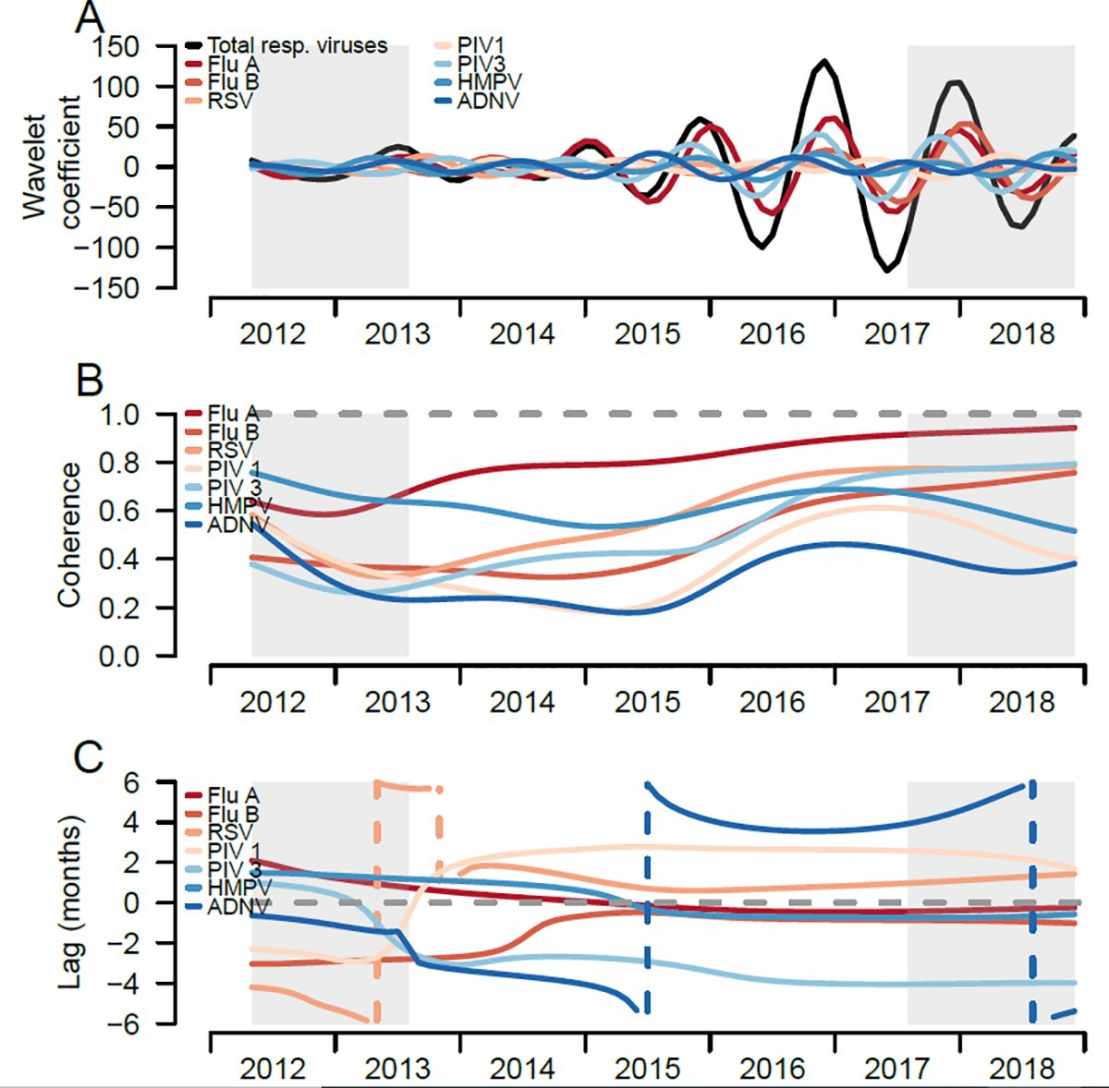
Trends of respiratory viral infections of pediatric participants, Sentinel Enhanced Dengue Surveillance System (SEDSS), Southern Puerto Rico, 2012–2018. A) Reconstructed seasonal patterns of monthly respiratory viral infections using the wavelet coefficient, total and individual respiratory viruses. Seasonal period was defined 8 to 16 months. B) Coherence comparing the similarity between the individual respiratory viruses patterns and the total respiratory infections. Dashed line at 1 refers to identical seasonal patterns and coherence at 0 refers to no association of two seasonal time-series. C) Phase differences comparing the timing of individual to total respiratory infections. Dashed line at 0 refers to patterns of individual and overall respiratory infections occurring at the time (i.e. no difference in phase). Grey shading reflects the cone of influence, where edge-effects of the time-series become important.

We compared the seasonal patterns of individual respiratory infections with that of all respiratory infections combined, a proxy for ILI, to distinguish how individual infections circulate compared to the ILI seasonal pattern. When comparing the seasonal components from the wavelet decompositions of individual respiratory infections and all respiratory infections combined, IAV has a similar seasonal pattern to the seasonal pattern of all respiratory infections combined (Fig 4A), with peaks in the winter months (i.e. December to January), and troughs in the spring and summer months (i.e. April to June). IBV, PIV 1, HRSV and HMPV shared similar seasonal patterns over time, whereas adenovirus had the most dissimilar pattern compared to other respiratory infections, peaking in the spring-summer months (**Fig 4A**).

Coherence analyses quantified the similarity of seasonal patterns of each respiratory infection compared to all respiratory infections; IAV (0.80), HMPV (0.62), and HRSV (0.59) had the highest mean coherence with the overall respiratory pattern (Table 3), and IAV and HRSV increased in coherence over the time-series (**Fig 4B**). HMPV had high coherence earlier in the time series and varied over time. In contrast, the mean coherence of IBV, PIV 1 and 3, and AdV ranged between 0.3 and 0. 5, and had lower, though varied, coherence to all respiratory infections over time.

**Fig 5:**
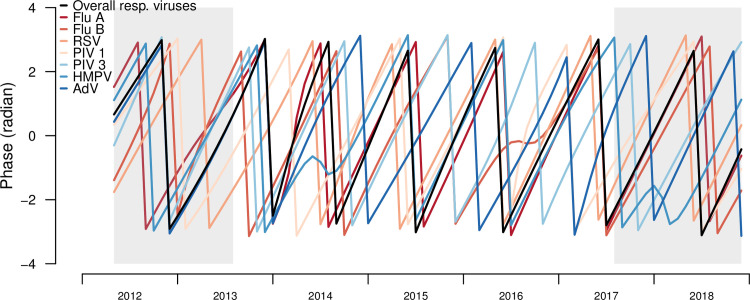
Phase angles of seasonal cycles of total and individual respiratory infections, Sentinel Enhanced Dengue Surveillance System (SEDSS), Southern Puerto Rico, 2012–2018.

Phase analyses quantified the timing of seasonal patterns of each respiratory infection compared to all respiratory infections (reference group). From the overall and individual respiratory infections phase angles, most infections shift their timing around the seasonal phase angles of overall respiratory infections (**Fig 5)**. From the phase differences, IAV had the most similar timing to the overall respiratory infections (**Fig 4C**). HRSV, PIV 1 and AdV viruses shifted their timing the most, with monthly infections lagging the overall infections in earlier years and then leading the overall infections in later years (Fig 4C). IAV had the highest similarity in timing with overall infections, with an estimated mean phase lag of 0.1 months, followed by HMPV (0.2 months) and HRSV (1.0 months). IBV lagged behind overall infections earlier in the time-series and became more synchronized over time. PIV 3 consistently lagged behind overall infections, with mean lag of 3 months (Table 3).

## Discussion

In this study, about half of pediatric patients with AFI had a viral or bacterial pathogen identified. Even with the extensive testing performed, determining an etiology was not possible in 52% of patients. A respiratory virus was found in one third of all patients tested and influenza A virus was the most common pathogen detected in those with an etiology identified. Arboviruses are an important cause of fever in children over 5 years old during defined outbreak periods. We also found differences by age with influenza virus being more common in older children while other respiratory viruses were more commonly identified in younger children. Finally, from longitudinal AFI surveillance and by isolating the seasonal cycles of the infection groups using time-series analyses, we found distinct seasonality for arboviruses (peaks in summer and early fall) and respiratory viruses (peaks in fall and winter) in a tropical setting, which supports growing evidence that respiratory infections exhibit seasonality, even in hotter and more humid climates, as opposed to constant year-round incidence [30–33].

Overall, influenza was the most common viral pathogen we identified, and accounted for over a quarter of the cases with a viral etiology identified, highlighting the importance of recognizing influenza as an important cause of fever and viral pediatric infections in Puerto Rico. Reports in other geographical areas, including Asia, Europe and Africa [3,15,34] have identified influenza as the most common viral etiology of AFI, but little data has been available for Puerto Rico and the Caribbean. Influenza vaccine coverage estimates among children 6 months through 17 years in Puerto Rico for the 2016–2017 influenza season were less than 50% for all age groups [35]. As vaccination is the main prevention strategy for influenza infection and its potential complications, strategies to improve vaccination coverage in this population could be implemented to reduce hospital visits due to AFI and hospitalization rates among all pediatric age groups.

In Puerto Rico, where emerging and reemerging arboviral diseases pose a permanent risk [36], arboviruses should be suspected in febrile children, particularly in the summer and fall months, from July to October. We found a trend of increasing proportion of dengue, Zika, and chikungunya diagnosis with increasing age. It is important for clinicians to suspect arboviruses infections in children of all ages in endemic areas, but especially in those older than 5 years old. Several factors should guide clinician diagnostic suspicion in a febrile child, including the local epidemiology. Clinicians should be aware of any ongoing outbreak or increase of number of cases for fever-causing diseases and use this information during the evaluation of patients in the pediatric ED. Clinicians should also consider the distinct seasonality of respiratory viruses and arboviruses when considering possible differential diagnoses. For diseases like dengue, that can be life-threatening, for which a specific treatment doesn’t exist, and vaccines are not yet available in Puerto Rico yet, improved clinical suspicion based on the seasonality found in AFI surveillance studies like ours can have important effects on the clinical outcome of pediatric patients, as early in-patient observation and intervention with adequate supportive treatment has been shown to reduce mortality [37].

Our analysis is one of few to quantify the differences in timing between the two main etiologic causes of AFI in Puerto Rico, and to show there are distinct seasonal patterns for pediatric respiratory and arboviral infections, particularly in a tropical setting. Arboviral peaks corresponded to major outbreaks and introduction of the new individual arboviruses (i.e. CHIKV and ZIKV), while seasonal peaks of individual respiratory infections varied in their timing. As expected, dengue infections peaked in summer and early fall, in the months that correspond to the rainy season in Puerto Rico (May to November) and that have been previously associated with an increase in dengue incidence [38]. After the 2016 Zika outbreak, arboviral infections remained low with smaller peaks of cases occurring throughout 2017–2018. Seasonal arboviral and respiratory infections are relatively asynchronous, with coherence varying over time, and having long lags (3.8 months) in timing of their patterns. The abnormal summer-month respiratory outbreaks of 2013 likely impacted the lower coherence in 2013–2014, with coherence increasing over later years until 2017. Similarly, the decreasing coherence from 2017–2018 is likely due to low arboviral incidence in 2017–2018.

In temperate zones, IAV, IAB and HRSV have very well-defined seasonality, being considered winter viruses, while AdV, HMPV, and PIV viruses circulate all year round with specific peaks for AdV subtypes in the fall (AdV1) and the spring-summer (AdV3) [32,39,40]. In our study, respiratory infections among pediatric participants generally peaked in the winter months, with IAV and IBV, HRSV, and AdV contributing the most to the overall infections. IAV was the most similar in frequency and timing to the overall respiratory infection patterns, followed by HMPV. Seasonal peaks varied for each respiratory pathogen, though some infections, such as IAV and HMPV, shared similarity in timing. AdV had the asynchronous pattern compared to overall infections, peaking in the spring and summer months. Though HRSV had high coherence with the overall respiratory pattern, it was more varied in its timing compared to the pattern of overall infections. For tropical and subtropical regions, previous reports have found similar patterns to those found in this study, with year- round incidence of respiratory viruses, and the presence of seasonal peaks. Most commonly, HRSV seasonal peaks have been reported in the rainy seasons and cooler seasons, respectively, with overlapping seasonality with influenza viruses peaks [34,39,41]. However, there are specific variations in the timing of the seasonal peaks by each place, that can be helpful to guide clinical suspicion for local health providers. For example, in Vietnam, a country with a tropical monsoon climate with high humidity, studies have found seasonal peaks of IAV during April to June [30], while in our study IAV peaked during December to January. In Egypt, a recent study showed that HRSV activity started in early winter and finished in early spring, similar to our findings, and found distinct annual peaks of HRSV that varied in duration and magnitude across the six tropical and subtropical countries included in their analysis [39]. They also found variation among participant countries regarding the timing of the peaks for influenza viruses and HRSV.

A recent study comparing the seasonality of influenza viruses in all ages in Puerto Rico to the seasonality in United States Health and Human Services regions, using data from SEDSS, found that in recent years synchronization for both IAV and IBV in both places has increased and suggested that factors like travel and United States influenza viral introductions to Puerto Rico can play an important role in such synchronization [42]. After the restrictions in travel, local mobility, and social gatherings related to the coronavirus disease 2019 (COVID-19), changes in respiratory viruses’ trends have started to be noticed [43]. Changes in dengue transmission dynamics have been reported in other countries like Thailand, with additional cases being expected as a result of the mobility restrictions in place [44]. Additional analyses of data after the implementation of COVID-19 related restrictions will be needed in Puerto Rico to establish their effect in the transmission of arboviruses and respiratory viruses in the island.

Our study had several limitations. First, the study focused diagnostic testing on arboviruses and respiratory viruses, leaving out systematic testing for parasitic, bacterial, or other viral etiologies. While arboviruses and respiratory viruses are clearly important pathogens for AFI in this population, we didn’t find a clear etiology in 52% of enrolled participants, and other types of pathogens should also be studied further in order to have a comprehensive understanding of AFI etiology. Second, this study includes only patients enrolled in one geographical area of Puerto Rico and the results are not necessarily generalizable to the rest of the island. Since CHIKV and ZIKV were introduced later in our time-series (2014–2015, and 2016), we could not evaluate their seasonal coherence and phase patterns. Additional years of surveillance will help to elucidate their patterns in the future.

With the wide array of etiological agents, the similar presentation in young children, and the high proportion of patients where all testing performed is negative, the accurate and opportune diagnosis of causes of AFI is challenging in most clinical settings. The lack of resources to conduct extensive testing, especially in areas like Puerto Rico where arboviruses are endemic, and the distinction between arboviral and other viral etiologies that can have important practical significance, highlights the need for the development of affordable, quick, and reliable, point-of-care tests for the most common causes of AFI.

The distinct seasonal patterns of respiratory viruses and arboviruses and differences by age groups seen in this study can guide clinical and laboratory assessment in pediatric patients with AFI in Puerto Rico. Additionally, our findings can help inform public health policies related to the vaccination target populations and timing, not only for existing vaccines like the influenza vaccine, but for potential vaccines to be introduced like the HSRV vaccine.

The patterns found highlight the continuous need for comprehensive AFI surveillance studies that can provide valuable information on the etiology and seasonality of acute febrile illness among pediatric populations and help clinicians and public health professionals guide their diagnostic process and disease control efforts.
